# Identification of Rare Variants in Right Ventricular Outflow Tract Obstruction Congenital Heart Disease by Whole-Exome Sequencing

**DOI:** 10.3389/fcvm.2021.811156

**Published:** 2022-01-24

**Authors:** Yue Zhou, Kai Bai, Yu Wang, Zhuo Meng, Shuang Zhou, Shiwei Jiang, Hualin Wang, Jian Wang, Mei Yang, Qingjie Wang, Kun Sun, Sun Chen

**Affiliations:** ^1^Department of Pediatric Cardiology, Xinhua Hospital, School of Medicine, Shanghai Jiao Tong University, Shanghai, China; ^2^Department of Pediatric Cardiology, The Second Affiliated Hospital & Yuying Children's Hospital of Wenzhou Medical University, Wenzhou, China

**Keywords:** whole-exome sequencing, congenital heart disease, right ventricular outflow tract obstruction, pulmonary valvular stenosis, pulmonary atresia with intact ventricular septum, single nucleotide polymorphism

## Abstract

**Background:**

Pulmonary atresia (PA) is a kind of congenital heart disease characterized by right ventricular outflow tract obstruction. It is divided into PA with intact ventricular septum (PA/IVS) whose favorable form is pulmonary valvular stenosis (PS), and PA with ventricular septal defect (PA/VSD) whose favorable form is tetralogy of Fallot (TOF). Due to limitations in genetics etiology, whole-exome sequencing (WES) was utilized to identify new variants associated with the diseases.

**Methods:**

The data from PS-PA/IVS (*n* = 74), TOF-PA/VSD (*n* = 100), and 100 controls were obtained. The common sites between PS and PA/IVS, PA/VSD and TOF, were compared. The novel rare damage variants, and candidate genes were identified by gene-based burden analysis. Finally, the enrichment analysis of differential genes was conducted between case and control groups.

**Results:**

Seventeen rare damage variants located in seven genes were predicted to be associated with the PS through burden analysis. Enrichment analysis identified that the Wnt and cadherin signaling pathways were relevant to PS-PA/IVS.

**Conclusion:**

This study put forth seven candidate genes (*APC, PPP1R12A, PCK2, SOS2, TNR, MED13*, and *TIAM1*), resulting in PS-PA/IVS. The Wnt and cadherin signaling pathways were identified to be related to PS-PA/IVS by enrichment analysis. This study provides new evidence for exploring the genetic mechanism of PS-PA/IVS.

## Introduction

Congenital heart disease (CHD) is the most common congenital disorder that affects about 1% liveborn infants (1). The incidence of CHD among live birth in China is ~0.9% (2). Pulmonary atresia (PA), a rare malformation of complex cyanotic CHD characterized by right ventricular outflow tract obstruction (RVOTO), accounts for about 1.3–3.4% of all heart abnormalities (3). The definition of PA is that there is no direct communication between the ventricle and the pulmonary blood flow. PA is traditionally divided into two groups: PA with intact ventricular septum (PA/IVS) and PA with ventricular septal defect (PA/VSD) (4). The characteristics of PA/IVS are membranous or muscular atresia of the RVOT without communication between the ventricle and pulmonary vessel (5). The pulmonary valvular stenosis (PS) might be a favorable form of PA/IVS. It is also a cyanotic CHD and comprises 8–12% of all CHDs (6, 7). PA/VSD is another group of PA and is considered as the extreme form of tetralogy of Fallot (TOF) (8). TOF is a heart defects syndrome with heart malformations of TOF are VSD, right ventricular hypertrophy, variable obstruction of the right ventricular outflow tract, and overriding aorta (9, 10). Patients born with these diseases might need to relieve RVOTO by intervention or surgery (11, 12). Although these diseases belong to or are similar to the PA family, they differ in structural abnormalities, hemodynamics, intervention strategies, and prognosis, implying varied pathogenesis (3).

Single-gene disorders and chromosomal anomalies could be the genetic etiology of CHD (13). Approximately 25% of CHDs could be explained based on genetic mutations (14). CHD patients with existing family history or suspected with other birth abnormalities, intellectual disability/developmental delay may undergo the clinical genetics evaluation; however, such patients constitute a small proportion. The majority of CHDs are isolated, sporadic, and non-syndromic cases (15). Due to the limitations in genetics etiology, current studies have utilized whole-exome sequencing (WES) to identify variants associated with the disease. Currently, known genes associated with PA/IVS include *HNRNPC, DANH10, GAJ1*, and *GDF1* (3, 16), while the genes related to PA/VSD are *GAJ5, MTHFR, MYH6, DANH1, PPP4C, FLT4, RICTOR*, and *FGF22* (16, 17). For sporadic TOF, *GATA4, NKX2.5, JAG1, FOXC2*, and *TBX1* are correlated with its genetic etiology (18). A previous study reported a plausible gene-disease association between TOF/PS and *CDC42BPA* and *FGD5* (19). Another study found that the pathogenesis of PS and PA/IVS might be related to the increased percentage of homozygous TT genotypes of *MTHFR* (20). However, only a few studies identified the rare variants of PS. The genetic etiology of PS and the differences in the genetic pattern between PS and PA are yet to be elucidated. In this study, we compared the genetic etiology of the four diseases according to the WES. In addition, we analyzed the potentially pathogenic genes of PS and PA/IVS.

## Materials and Methods

### Study Population

**Figure 1:**
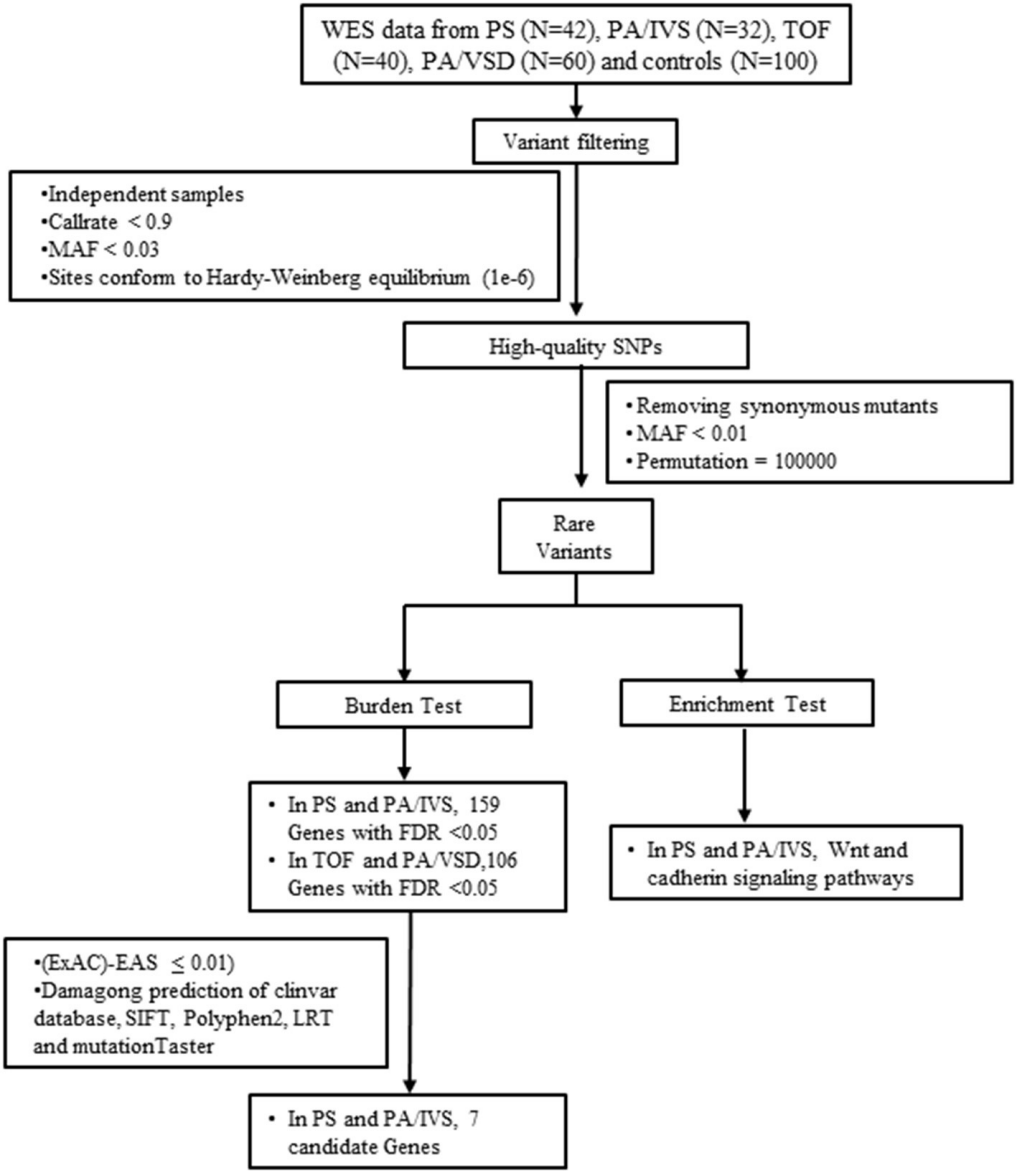
The flow chart showed the different steps taken during whole-exome sequencing analysis. After variant calling, annotation and screening, variants were filtered by gene-based burden analysis. Candidate genes were collected by gene expression analysis. FDR, false discovery rate; MAF, minor allele frequency; PA/IVS, pulmonary atresia with intact ventricular septum; PA/VSD, pulmonary atresia with ventricular septal defect; PS, Pulmonary valvular stenosis; SNP, single nucleotide polymorphism; TOF, tetralogy of Fallot; WES, whole-exome sequencing.

The flow chart of the study was illustrated in Figure 1. The cohort comprised 174 sporadic patients diagnosed with CHD by echocardiography and 100 healthy controls. The cohort comprised Chinese Han individuals aged 0–14-year-old with no family history of CHD. The CHD cases were further divided into four groups: PS (*n* = 42), PA/IVS (*n* =32), TOF (*n* =40), and PA/VSD (*n* =60) (Supplementary Table 1). The patients were selected according to the inclusion criteria that included patients with neither other CHDs nor identified syndromic or chromosomal disorders. The study was approved by the Ethics Committee of Xinhua Hospital affiliated with Shanghai Jiao Tong University (XHEC-C-2019-083), and conducted in accordance with the Declaration of Helsinki. Informed consent was obtained from all participants' parents.

### WES

Peripheral blood DNA was extracted using the QIAamp^TM^ DNA and Blood Mini Kit (Qiagen, Germany) following the standard instructions. The DNA concentration and quality were assessed by measuring the 260/280 optical density value on a NanoDrop 2000c spectrophotometer (Thermo Fisher Scientific, USA). WES was performed using the Agilent Sure Select Target Enrichment kit (V6 58 Mbp; Agilent Technologies, USA) for sequence capture and Illumina HiSeq2500 for sequencing (Illumina, USA).

### Reads Mapping, Variant Calling, and Annotation

Sequencing reads were mapped to the Genome Reference Consortium Human Genome Build 37 (GRCh37). Single nucleotide variant (SNV) and Insert/Delete (Indel) results were processed simultaneously by Genome Analysis Toolkit (GATK) HiplotypeCaller. Subsequently, ANNOVAR software was employed to complete the mutation annotation, including the population control databases (ExAC, genomAD, 1000 Genomes Projects, etc.) and mutation prediction programs (SIFT, Polyphen-2, and MutationTaster, etc.).

### Single Nucleotide Polymorphisms Screening

Original data should be controlled to remove the sites with low detection rate, low allele frequency, and not in agreement with the Hardy–Weinberg equilibrium. The details are as follows:

(1) Duplicate samples and related samples were removed.
(2) Callrate ≥ 0.9.
(3) Minor allele frequency (MAF) ≥ 0.03.
(4) Remove sites that do not conform to Hardy-Weinberg equilibrium (1e-6).

### Burden Analysis

This study used the “T1” criterion: if a variation site (SNV or Indels) had a minor allele frequency (MAF) <0.01, then the variation site was regarded as a rare variant site (21).


$$\begin{array}{l} \text{MAF}=\frac{\text{the}\;\text{number}\;\text{of}\;\text{M}\text{As}}{2*\text{the}\;\text{number}\;\text{of}\;\text{samples}} \end{array}$$

After removing synonymous mutations, the non-synonymous mutations in rare mutations were divided into five groups: (1) non-synonymous rare mutations (non-synonymous sets); (2) deleterious rare mutations labeled as “deleterious” by Polyphen-2 humdiv (deleterious sets); (3) these rare mutations labeled as nonsense, Indel frameshift, and splice site (disruptive sets); (4) deleterious rare mutations labeled as “deleterious” in at least one of the five protein prediction algorithms, including LRT Score, MutationTaster, Polyphen-2 humdiv, Polyphen-2 humvar, and SIFT (Broad deleterious sets); (5) rare mutations labeled “deleterious” by five protein prediction algorithms (Strict deleterious sets) (21).

In this study, a variant of the Combined Multivariate Collapsing test, which grouped the count of alleles of SNVs in cases and controls, was performed. To assign a statistical significance, we permuted the phenotype labels 100,000 times. Association analysis was performed using PLINK/SEQ. Frequency-weighted test (FW) and variable threshold test (VT) methods were used to calculate the correlation between rare mutations in each gene region and the diseases (21).

### Rare Variants Validation

Furthermore, these candidate variants were confirmed by Sanger sequencing. The primers for PCR amplification were designed using Primer premier5; the sequences were listed in Supplementary Table 2.

## Results

### Overview of SNPs

The obtained data were filtered by total sample variation screening, callrate (missing rate) test, singleton (defined as a variant present in only one sample) test, heterozygosity test, and Ti/Tv ratio test. Based on the above quality control methods, one control sample was removed due to many singletons, which indicated the low quality of DNA. Another control sample was removed because of high heterozygosity (Supplementary Figure 1).

**Figure 2:**
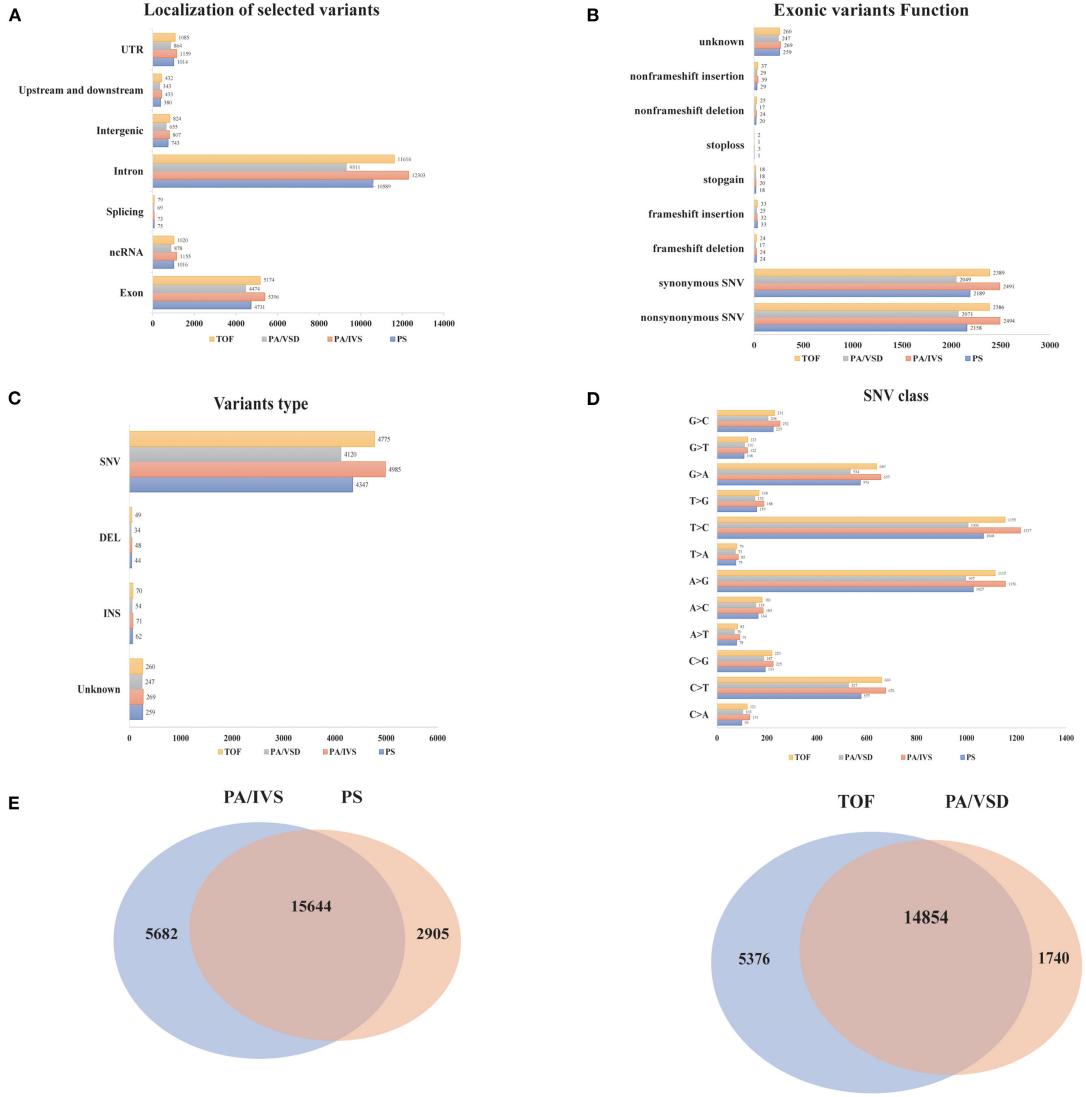
The comparisons of the variants among four disease groups. The localization of selected variants **(A)**, variants type **(B)**, the function of exonic variants **(C)**, and SNV classes **(D)** of four diseases. **(E)** The comparisons of the variants between PA/IVS and PS, TOF and PA/VSD.

We compared the common and different SNPs between PS and PA/IVS, TOF and PA/VSD. After quality control, 18549, 21326, 16594, and 20230 SNPs and Indels were identified in PS, PA/IVS, PA/VSD, and TOF, respectively. Next, we counted and compared the localization of selected variants, variants type, the function of exonic variants, and SNV classes of four diseases (Figures 2A–D). The most selected variants were in introns, followed by exons. Most of the exonic variants were SNVs, and the number of Inserts (INSs) and Deletes (DELs) was small. Regarding the exonic function, the number of synonymous and non-synonymous SNVs were significantly larger than the others (non-frameshift INS/DEL, stoploss, stopgain, frameshift INS/DEL). In these SNVs, T>C was the most common class. The number of common sites between PS and PA/IVS was 15644, while those between PA/VSD and TOF were 14854 (Figure 2E). A previous study speculated that PA/IVS was the extremely severe form of PS, and PA/VSD, another group of PA, was the extreme form of TOF (6–8). Because of the limited sample size of each disease, we combined PS and PA/IVS as one group PS-PA/IVS, and PA/VSD and TOF as another group TOF-PA/VSD for further analysis.

### Rare Variants Analysis

We conducted a principal component analysis (PCA) between these two groups with control and found that the distribution of disease and control samples was consistent (Supplementary Figures 2A,B).

### Burden Test

The definition of rare variants was that the MAF of variants was <1% or <0.5% (22). In order to conduct the correlation analysis on the rare mutants, after setting MAF <0.01, we conducted the burden test of rare variants undergoing selection and classification. To enrich the harmful alleles, we considered three groups of variants (non-synonymous, deleterious and disruptive sets) (21). Given the threshold of *p* = 0.01, 21, and 10 genes with potential pathogenicity of PS-PA/IVS were found in the non-synonymous and deleterious sets, respectively; 20 and 9 genes with potential pathogenicity of TOF-PA/VSD were found in the non-synonymous and deleterious sets, respectively; no gene in two diseases was found in disruptive sets. Since only a few studies have assessed the rare variants of PS-PA/IVS, we mainly analyzed the genes with potential pathogenicity of the two diseases. A total of 26 genes with potential disease pathogenicity were identified (Table 1). 218 unique non-synonymous or splice-site SNVs or Indel frameshifts with MAF <0.01 were identified among these genes. Also, 26 alternate allele counts in cases and 19 alternate allele counts in controls were observed.

**Table 1:** A list of genes with the potential pathogenicity of disease were identified by burden test.

**Disease**	**Gene**	**Chr**	**N (variants)**	**N (cases/controls)**	**Cases**	**Controls**	**% Freq (cases)**	**% Freq (controls)**	***P***	**OR**
PS-PA/IVS	*PIEZO1*	16	45	74/100	18	10	24.32	10.00	0.0011	1.3
	*C7*	5	8	74/100	6	0	8.11	0.00	0.0015	-
	*TH*	11	7	74/100	6	0	8.11	0.00	0.0016	-
	*GBF1*	10	13	74/100	8	2	10.81	2.00	0.0035	3
	*COBLL1*	2	10	74/100	7	1	9.46	1.00	0.0035	5.2
	*APC*	5	12	74/100	8	2	10.81	2.00	0.0038	3
	*TTC22*	1	7	74/100	6	0	8.11	0.00	0.0043	-
	*CACNA1H*	16	30	74/100	17	10	22.97	10.00	0.0043	1.3
	*ETV4*	17	5	74/100	5	0	6.76	0.00	0.0044	-
	*SLFN14*	17	8	74/100	5	0	6.76	0.00	0.0048	-
	*FAT2*	5	35	74/100	15	8	20.27	8.00	0.0049	1.4
	*GRIK1*	21	6	74/100	5	0	6.76	0.00	0.0049	-
	*NEK1*	4	11	74/100	5	0	6.76	0.00	0.0050	-
	*MCF2L*	13	16	74/100	11	4	14.86	4.00	0.0050	2
	*FDFT1*	8	7	74/100	8	1	10.81	1.00	0.0060	5.9
	*PLEKHG4B*	5	21	74/100	12	6	16.22	6.00	0.0071	1.5
	*SREK1*	5	8	74/100	7	1	9.46	1.00	0.0072	5.2
	*HELQ*	4	10	74/100	6	1	8.11	1.00	0.0072	4.4
	*SAP130*	2	5	74/100	6	1	8.11	1.00	0.0076	4.4
	*PHF3*	6	8	74/100	6	1	8.11	1.00	0.0078	4.4
	*CCT6B*	17	5	74/100	6	1	8.11	1.00	0.0082	4.4
	*AHI1*	6	12	74/100	8	3	10.81	3.00	0.0083	2
	*WDR73*	15	6	74/100	7	2	9.46	2.00	0.0092	2.6
	*CHPF2*	7	10	74/100	7	2	9.46	2.00	0.0096	2.6
	*GRAMD1A*	19	11	74/100	8	3	10.81	3.00	0.0096	2
	*SEC14L5*	16	8	74/100	8	3	10.81	3.00	0.0098	2

*Chr, chromosome; PA/IVS, pulmonary atresia with intact ventricular septum; PA/VSD, pulmonary atresia with ventricular septal defect; PS, Pulmonary valvular stenosis; TOF, tetralogy of Fallot*.

**Figure 3:**
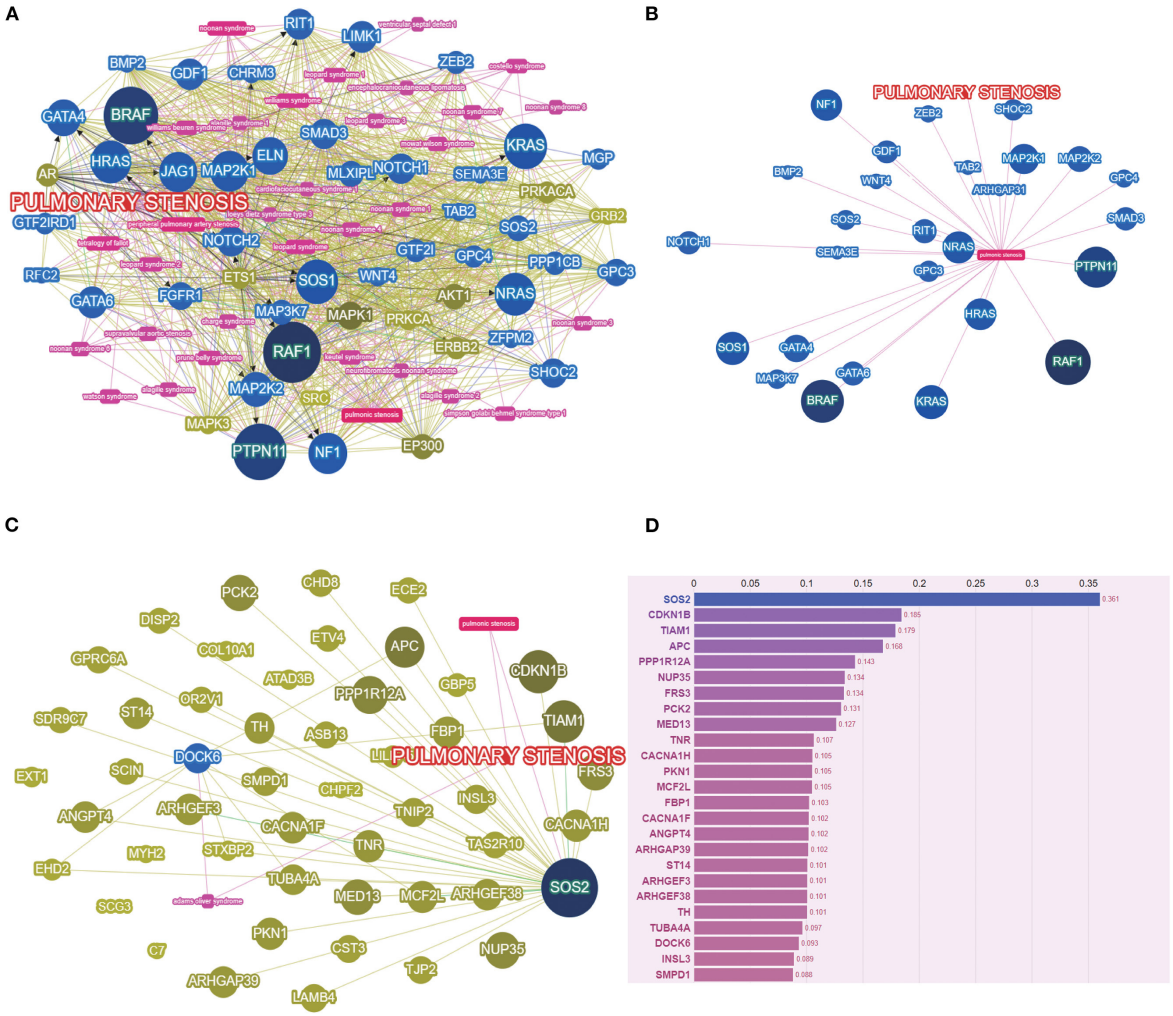
The PS-associated genes were identified by gene-based burden analysis. **(A)** The known genes related to the PS phenotypes were searched and summarized. **(B)** Genes associated with PS phenotypes were shown after excluding the genes related to the syndromes. **(C)** The association between genes tested and disease phenotype was predicted. **(D)** The score of genes tested was listed by Phenolyzer (http://phenolyzer.wglab.org/).

The variant sets based on multiple protein prediction algorithms might yield strong association signals. Therefore, in exploratory analyses, we investigated two additional variant sets (broad deleterious and strict deleterious sets) (21). The threshold was set to *p* = 0.05 for these two sets, following which, we identified 159 genes of PS-PA/IVS and 106 genes of TOF-PA/VSD. Next, we searched and summarized the known genes related to the PS phenotypes (Figure 3A; http://phenolyzer.wglab.org/). After excluding the genes related to the syndromes, only those associated with the PS phenotype remained (Figure 3B). Then, we predicted the association between genes tested in this study and disease phenotype (Figure 3C) and identified two seed genes, *SOS2* and *DOCK6*. A seed gene meant that this gene was directly correlated to the input term based on the existing databases. *SOS2* was a known gene related to the PS phenotype, while *DOCK6* was associated with Adams–Oliver syndrome, whose clinical performance might exist PS phenotype. Next, we listed the first 25 genes in Figure 3D. Except for the two seed genes, the remaining genes, such as *CDKN1B, TIAM1, APC*, and *PPP1R12A*, were also predicted to be relevant to disease phenotypes by gene-gene linkage (Figure 3D).

### Enrichment Analysis

**Figure 4:**
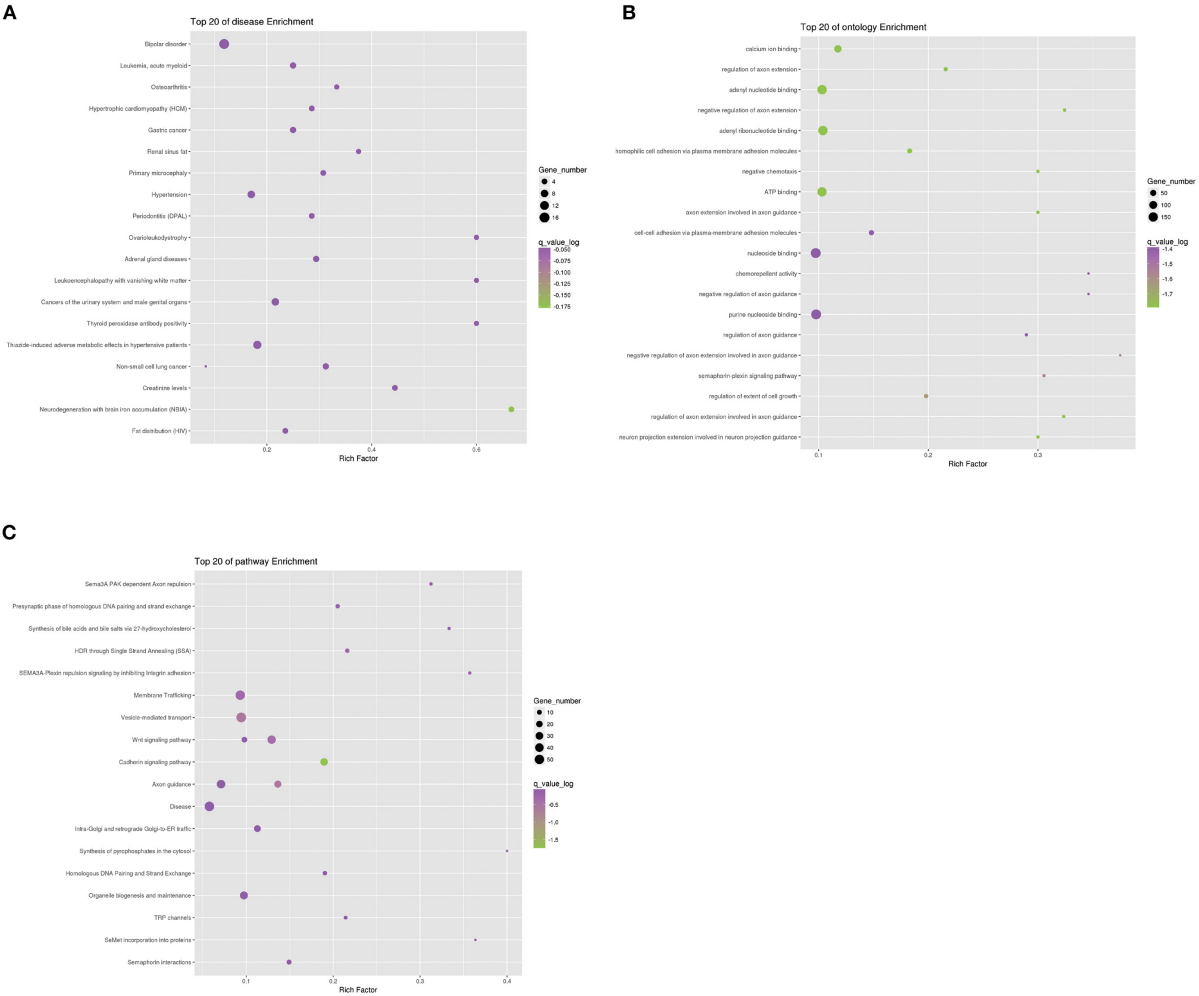
Enrichment analysis of differential genes between PS-PA/IVS and control groups. Top 20 of disease **(A)**, ontology **(B)**, and pathway **(C)** enrichment were listed.

To further analyze the function of differential genes between case and control groups, we conducted the enrichment analysis of genes in strict deleterious set. The enrichment analysis mainly included disease, ontology, and pathway enrichment (Figure 4). Regarding disease enrichment, these genes were involved in a variety of systemic diseases, among which cardiovascular diseases mainly included hypertrophic cardiomyopathy and hypertension. In addition, pathway enrichment found Wnt and cadherin signaling pathways. The Wnt signaling pathway was a highly conserved regulatory pathway during embryonic development. When dysregulated, it could result in congenital malformations, including aortic valve stenosis (23, 24). The cadherin signaling pathway also played a role during aortic valve maturation (25). However, whether these pathways affect PS or PA/IVS was yet to be explored. The enrichment analysis might be limited by fewer pathogenic genes associated with PS and PA/IVS.

### Rare Variants Validation

**Figure 5:**
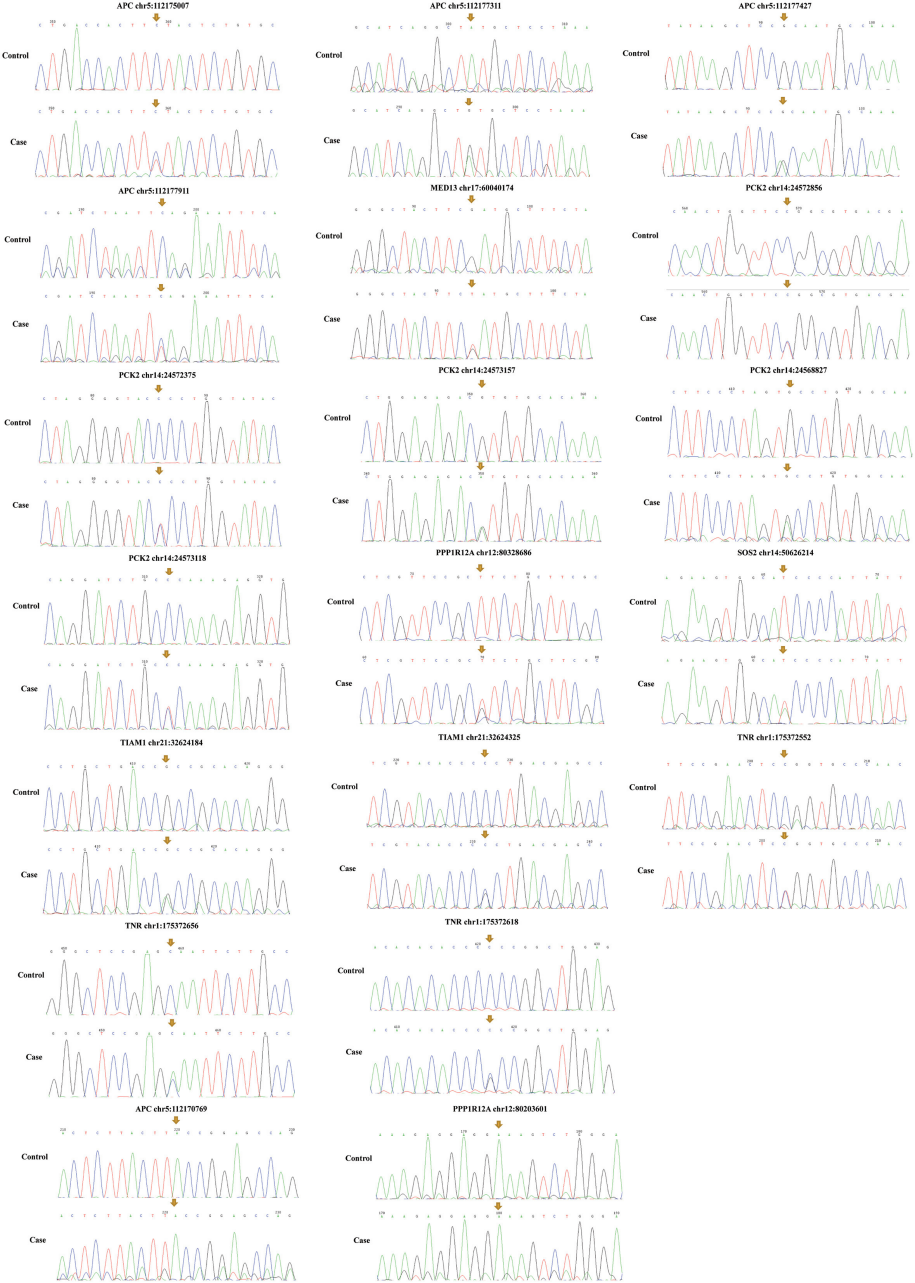
Mutation validation of seven genes with 19 SNPs. Only two SNPs (*APC* chr5:112170769 and *PPP1R12A* chr12:80203601) were not validated successfully. The yellow arrows represent the sites of SNPs.

Among the above 159 selected genes of PS-PA/IVS, we selected the first ten genes for further analysis. After being predicted to affect the protein function according to ClinVar database, SIFT, Polyphen-2, LRT, and MutationTaster prediction, 19 SNPs located in seven genes, *SOS2, TIAM1, APC, PPP1R12A, PCK2, MED13, and TNR* were left (Table 2; Supplementary Table 3). Thus, seven genes with 19 SNPs were validated. Next, according to the position of SNPs, we designed primers to validate the mutations by Sanger sequencing. We found that only two SNPs were not validated successfully (*APC* chr5:112170769 and *PPP1R12A* chr12:80203601) (Figure 5). However, whether these genes were related to phenotypes needs to be substantiated further by clinical studies.

**Table 2:** Rare variant of seven candidate genes associated with PS-PA/IVS.

**Chromosome**	**Position**	**Base Change**	**Gene**	**AAChange**	**TxChange**	**ExAC-EAS**	**CLINSIG**	**SIFT**	**PolyPhen2**	**LRT**	**Mutation Taster**
5	112175007	G > A	*APC*	p.R1221K	c.3662G>A	0.0001	Uncertain	-	D	D	D
5	112177311	A > G	*APC*	p.Y1989C	c.5966A>G	0.0001	Uncertain	-	D	D	D
5	112170769	A > G	*APC*	p.Y604C	c.1811A>G	-	-	D	D	D	D
5	112177427	G > A	*APC*	p.A2028T	c.6082G>A	0.0001	Uncertain	-	D	D	D
5	112177911	C > T	*APC*	p.S2189L	c.6566C>T	0	Uncertain	-	D	D	D
17	60040174	C > A	*MED13*	p.R1668L	c.5003G>T	-	-	D	D	D	D
14	24572856	C > T	*PCK2*	p.R402W	c.1204C>T	0.0003	-	D	D	D	D
14	24572375	C > T	*PCK2*	p.P326L	c.977C>T	0.0007	-	D	D	D	D
14	24573157	G > A	*PCK2*	p.R502H	c.1505G>A	0.0001	-	D	D	D	D
14	24568827	G > A	*PCK2*	p.A305T	c.913G>A	-	-	D	D	D	D
14	24573118	C > T	*PCK2*	p.P489L	c.1466C>T	-	-	D	D	D	D
12	80203601	A > G	*PPP1R12A*	p.S390P	c.1168T>C	-	-	D	D	D	D
12	80328686	T > G	*PPP1R12A*	p.K9T	c.26A>C	-	-	D	D	D	D
14	50626214	A > T	*SOS2*	p.I596N	c.1787T>A	-	-	D	D	D	D
21	32624325	C > G	*TIAM1*	p.G382R	c.1144G>C	0.0005	-	D	D	D	D
21	32624184	C > T	*TIAM1*	p.A429T	c.1285G>A	-	-	D	D	D	D
1	175372552	G > A	*TNR*	p.R234W	c.700C>T	0.0002	-	D	D	D	D
1	175372656	C > A	*TNR*	p.C199F	c.596G>T	-	-	D	D	D	D
1	175372618	C > G	*TNR*	p.G212R	c.634G>C	0	-	D	D	D	D

## Discussion

After negative results in chromosomal microarray and karyotype testing, WES was a common method to diagnose the underlying genetic cause in 25–35% of children with an unexplained presumed genetic disease (such as a birth defect) (26). As it is the most common birth defect, the pathogenesis for CHD needs to be elucidated. Hitherto, only a few studies have specifically focused on PS or PA/IVS. In order to clarify the genetic cause underlying PS-PA/IVS, we adopted WES to identify the damaging variants and potential pathogenic genes in 74 PS-PA/IVS cases and 100 healthy controls. Further burden test and phenotype analysis identified seven candidate genes, including *SOS2, TIAM1, APC, PPP1R12A, PCK2, MED13, and TNR*. Enrichment analysis identified the Wnt and cadherin signaling pathways linked to PS. Due to the little-known genetic cause of PS-PA/IVS, the disease enrichment analysis enriched a variety of systemic diseases. However, additional basic and clinical studies are required to confirm whether these genes were pathogenic in PS-PA/IVS.

In order to identify the rare variants associated with PS-PA/IVS, we conducted the burden test and identified seven genes that might be related to the disease. Based on the analysis of a phenolyzer, *SOS2* was the seed gene of PS. A previous study found that *SOS2* was homologous to *SOS1*, the second gene frequently related to Noonan syndrome (27). It is the second most common syndromic cause of CHD, and its most common phenotype is PS (28). Noonan syndrome was caused by functional dysregulation in the ERK signal transduction pathway. A recent study demonstrated that *SOS2* mutation causes Noonan syndrome (29). However, in the current study, we found that the *SOS2* mutation might be a genetic cause of non-syndromic PS.

Except for the seed gene *SOS2*, the other six genes associated with PS were predicted and found to be involved in three pathways. The first pathway was Wnt signaling pathway and mainly included *APC* and *PPP1R12A* in this study. *APC*, a Wnt signaling pathway regulator, plays a critical role in cellular processes, including signal transduction and cell adhesion. Mutant mice and cells studies showed that *APC* inhibits the Wnt signaling pathway (30). Moreover, the signaling pathway has been proven to be critical for cardiac development. Canonical Wnt signaling pathway was associated with cardiac valve formation. The mutated *APC* protein would give rise to an excessive endocardial layer fused with the atrioventricular outflow tract and markedly expand the endocardial cushions through overexpressing β-catenin during EMT (31). Another gene related to the Wnt pathway was *PPP1R12A*, which was identified as a putative cofactor of *NKX2.5* (32). It encoded a regulatory subunit of myosin phosphatase and was related to significant roles in several cellular processes, such as gene expression regulation, cell cycle, and embryonic development (33). The knockdown of *PPP1R12A* enhanced cardiomyogenesis and rescued Wnt3a-mediated inhibition in mice embryonic stem cells (32).

The second pathway was the thyroid hormone (TH) signaling pathway, including only *MED13*. *MED13* was the major target of microRNA-208, which was specific to cardiac muscle, and modulated the activity of the thyroid hormone receptor related to cardiac hypertrophy. In cyanotic CHD children, adenosine-to-inosine RNA editing in *MED13* was significantly higher than in acyanotic patients (34), thereby implying that *MED13* might be involved in cyanotic CHD.

The last pathway was the PI3K-AKT pathway, including *TIAM1, PCK2*, and *TNR*. The triplication of the *TIAM1-Knj6* region was necessary to cause Down's syndrome-associated heart defects in mice (35). *TIAM1* was related to the non-syndromic mitral valve prolapse (36). A previous study found that *TIAM1* activates the PI3K pathway when hypertrophy is induced in neonatal rat cardiomyocytes (37). *PCK2* encoded the mitochondrial phosphoenolpyruvate carboxykinase. Increasing *PCK2* promotes hypertrophic growth in mice while knocking down the gene attenuates norepinephrine-induced cardiac hypertrophy (38). A recent study found that *PCK2* regulates the osteogenic capacity of mesenchymal stem cells through the AMPK pathway (39). However, the biological role of *PCK2* is not yet clarified. The Tenascin family modulates the cellular response to growth factors and cell adhesion. *TNR* is an extracellular matrix protein specific to the central nervous system (40). Strikingly, *TNR* and CHD have not been investigated previously. Nonetheless, *TNR* is involved in focal adhesion and PI3K-AKT signaling pathway (41, 42). All these genes were predicted to correlate with the phenotype of PS-PA/IVS; however, clinical and basic studies are still lacking.

## Limitations

Firstly, due to the small sample size, establishing a validation cohort to confirm the candidate genes was difficult. Secondly, because of the small sample size of each disease group, we combined PS and PA/IVS into one group for further rare variants analysis. Hence, fewer genes differed between the two groups. Lastly, the genetic background of patients was limited by the lack of parental samples.

## Conclusion

In conclusion, an effective analytical bioinformatics method allowed us to identify rare damage variants. Thus, this study put forth seven candidate genes (*APC, PPP1R12A, PCK2, SOS2, TNR, MED13*, and *TIAM1*), resulting in PS-PA/IVS, and which have not been reported in either animals or humans. Enrichment analysis identified the Wnt and cadherin signaling pathways linked to PS. This study provides new evidence for exploring the genetic mechanism of PS-PA/IVS. However, additional studies are required to verify these genes.

## Data Availability Statement

The datasets presented in this article are not readily available due to ethical and privacy restrictions. Requests to access the datasets should be directed to the corresponding author.

## Ethics Statement

The protocol was approved by the Ethical Committee of Xinhua Hospital (XHEC-C-2019-083). Written informed consent to participate in this study was provided by the participants' legal guardian/next of kin.

## Author Contributions

SC, KS, and QW contributed equally to the study and conceived and designed the study. YZ, KB, and YW prepared an analytical plan, analyzed data, and drafted the initial manuscript. ZM, SZ, and SJ were involved in sample collection. HW, JW, and MY collaborated in the revision and interpretation of the data and results. All authors reviewed and revised the manuscript, approved the final manuscript as submitted, and agreed to be accountable for all aspects of the work.

## Funding

This work was supported by the Key Program for International S&T Cooperation Projects of China (81720108003), the National Key R&D Program of China (2018YFC1002400 and 2018YFC1002403), and the National Natural Science Foundation of China (81800281).

## Conflict of Interest

The authors declare that the research was conducted in the absence of any commercial or financial relationships that could be construed as a potential conflict of interest.

## Publisher's Note

All claims expressed in this article are solely those of the authors and do not necessarily represent those of their affiliated organizations, or those of the publisher, the editors and the reviewers. Any product that may be evaluated in this article, or claim that may be made by its manufacturer, is not guaranteed or endorsed by the publisher.
