# Supplementary material for: Identification of Rare Variants in Right Ventricular Outflow Tract Obstruction Congenital Heart Disease by Whole-Exome Sequencing
Source: Front Cardiovasc Med. 2022 Jan 24;8:811156. doi: 10.3389/fcvm.2021.811156 (PMC8818757; doi:10.3389/fcvm.2021.811156)

Table S1. The baseline information including age and sex of patients recruited in this study

	PS	PA/IVS	TOF	PA/VSD	Control
Total (N)	42	32	60	40	100
Male (%)	26 (61.9)	19 (59.4)	23 (57.5)	39 (65)	65 (65)
Female (%)	16 (38.1)	13 (40.6)	17 (42.5)	21 (35)	35 (35)
Male-to-female ratio	1.63	1.46	1.35	1.86	1.86
Age (months)	11.48±4.00	16.81±4.83	11.76±1.93	53.27±5.45	56.83±4.34

* Data are expressed as the mean ± SEM. PA/IVS: pulmonary atresia with intact ventricular septum; PA/VSD: pulmonary atresia with ventricular septal defect; PS: Pulmonary valvular stenosis; TOF: tetralogy of Fallot.

Table S2. Sequence of primers

Gene	Gene Positation	Forward	Reverse
**APC**	chr5:112175007	TCTGGGTCTACCACTGAA	TGTCTGAGCACCACTTTT
**APC**	chr5:112177311	AACTAACCTCCAACCAAC	TATGCCACCCATATTTCT
**APC**	chr5:112170769	ATTTACCAGTGAGGGACG	CACCTATGGGCTACACCT
**APC**	chr5:112177427	CAGGCTATGCTCCTAAAT	TCACCTAATATGCCACCC
**APC**	chr5:112177911	GATTCCATCCTTTCCCTG	TTGGCTCATCTGTCTACCTG
**MED13**	chr17:60040174	TGATTCACATGCAGTCAC	ATTGCTTTCTCCTTTTCT
**PCK2**	chr14:24572856	GGGATAGCCGAGGTCTTA	CAGGTAGCTCCGAATGTC
**PCK2**	chr14:24572375	TGGGATAGCCGAGGTCTT	AGGGAGGAATGGTGAGGG
**PCK2**	chr14:24573157	ACTACCTGGAACACTGGC	GATAGGTGGGAAATGAAAA
**PCK2**	chr14:24568827	CTTTGCCCTACGCATCGC	CTGACAAGAGCCAGAACCA
**PCK2**	chr14:24573118	ACTACCTGGAACACTGGC	AAGATAGGTGGGAAATGAA
**PPP1R12A**	chr12:80203601	ACTGGCATTGTTCTTGTG	AGTCTGGGACTTGAAGCT
**PPP1R12A**	chr12:80328686	TGCCCTGTAGAGCCTTGCG	GCCATCGTCGAACTTCACCTT
**SOS2**	chr14:50626214	TGGGCAACAGAGCAAGAC	CCAAAGATGAGAACAGCA
**TIAM1**	chr21:32624325	GTGCCTTATGCGTATCCC	CACCCAGTAGTGCTTCCA
**TIAM1**	chr21:32624184	GTGCCTTATGCGTATCCC	CACCCAGTAGTGCTTCCA
**TNR**	chr1:175372552	CACTGCTGCTCTTTGACC	GTACCGTTGGCACATCTC
**TNR**	chr1:175372656	GCTGCTCTTTGACCTTCT	TTTGCCTAAACTTCACTTTCTC
**TNR**	chr1:175372618	GCTGCTCTTTGACCTTCT	TTTGCCTAAACTTCACTTTCTC

Table S3. A list of 7 candidate genes predicted to affect the protein function were identified by burden test.

Disease	Gene	Chr	N (variants)	N (cases/controls)	Cases	Controls	% Freq (cases)	% Freq (controls)	P	OR
**PS-PA/IVS**	*APC*	4	20	74/100	10	7	13.51	7.00	0.0305	1.1
	MED13	9	6	74/100	4	1	5.41	1.00	0.0465	3.0
	PCK2	8	9	74/100	6	2	8.11	2.00	0.0223	2.2
	PPP1R12A	5	5	74/100	3	0	4.05	0	0.0427	-
	SOS2	1	5	74/100	5	0	6.76	0	0.0139	-
	TIAM1	3	6	74/100	3	0	4.05	0	0.0419	-
	TNR	10	6	74/100	3	0	4.05	0	0.0416	-

Figure S1. Quality control screening of samples. The obtained data were filtered by total sample variation screening to remove the samples that deviated significantly from the entire population (A), callrate (missing rate) test to remove samples with many missing sites (B), singleton (defined as a variant present in only one sample) test to remove samples with a large number of singletons (C), heterozygosity test to remove the samples with high heterozygosity (D), and Ti/Tv ratio to determine the reliability of sample variations (E).

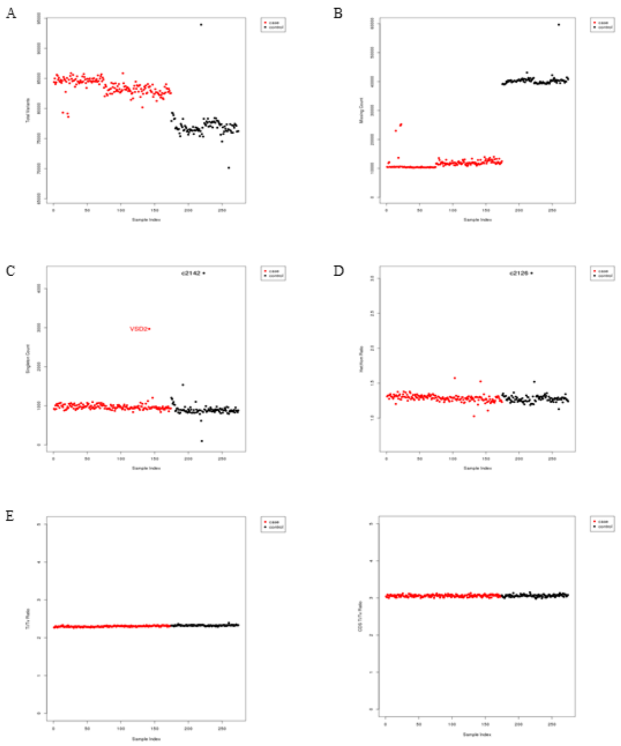

Figure S2. The SNP-based association analysis was conducted between PS-PA/IVS and TOF-PA/VSD groups with control group. (A) The principal component analysis (PCA) between PS-PA/IVS and control group. The red dots mean samples of PS-PA/IVS and the black dots mean samples of control group. (B) The PCA between TOF-PA/VSD and control group. The red dots mean samples of TOF-PA/VSD and the black dots mean samples of control group.

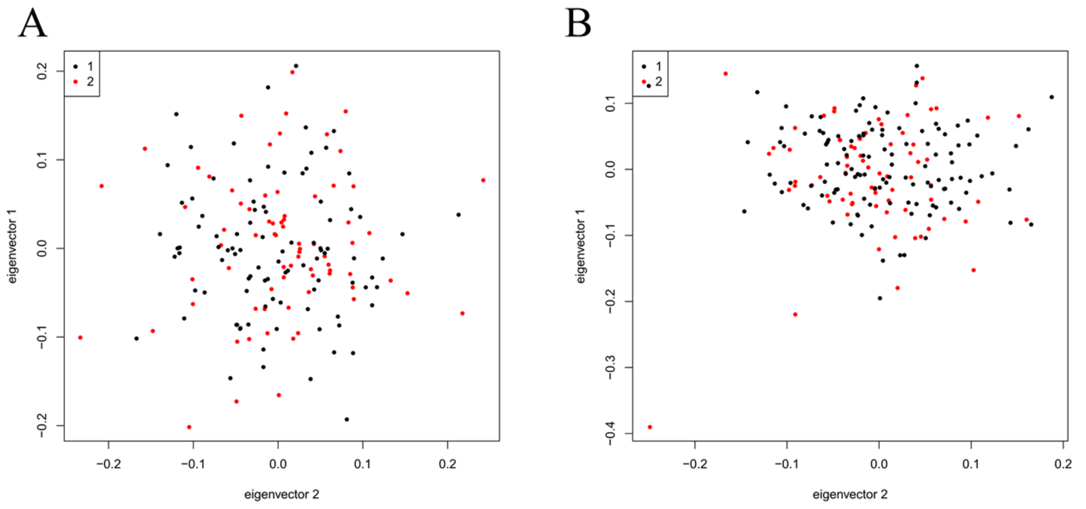

Supplement: Supplementary file 1 [file Data_Sheet_1.PDF]
